# Risk factors for recurrent wheezing in infants: a case-control study

**DOI:** 10.1590/S1518-8787.2016050005100

**Published:** 2016-04-15

**Authors:** Roberta Barros de Sousa, Décio Medeiros, Emanuel Sarinho, José Ângelo Rizzo, Almerinda Rêgo Silva, Ana Carolina Dela Bianca

**Affiliations:** I Programa de Pós-Graduação em Ciências da Saúde. Centro de Ciências da Saúde. Universidade Federal de Pernambuco. Recife, PE, Brasil; II Departamento Materno Infantil. Centro de Ciências da Saúde. Universidade Federal de Pernambuco. Recife, PE, Brasil; III Departamento de Medicina Clínica. Centro de Ciências da Saúde. Universidade Federal de Pernambuco. Recife, PE, Brasil

**Keywords:** Infant, Asthma, Risk Factors, Respiratory Sounds, Allergens, Smoke, Case-Control Studies

## Abstract

**OBJECTIVE:**

To evaluate the association between recurrent wheezing and atopy, the Asthma Predictive Index, exposure to risk factors, and total serum IgE levels as potential factors to predict recurrent wheezing.

**METHODS:**

A case-control study with infants aged 6-24 months treated at a specialized outpatient clinic from November 2011 to March 2013. Evaluations included sensitivity to inhalant and food antigens, positive Asthma Predictive Index, and other risk factors for recurrent wheezing (smoking during pregnancy, presence of indoor smoke, viral infections, and total serum IgE levels).

**RESULTS:**

We evaluated 113 children: 65 infants with recurrent wheezing (63.0% male) with a mean age of 14.8 (SD = 5.2) months and 48 healthy infants (44.0% male) with a mean age of 15.2 (SD = 5.1) months. In the multiple analysis model, antigen sensitivity (OR = 12.45; 95%CI 1.28–19.11), positive Asthma Predictive Index (OR = 5.57; 95%CI 2.23–7.96), and exposure to environmental smoke (OR = 2.63; 95%CI 1.09–6.30) remained as risk factors for wheezing. Eosinophilia ≥ 4.0% e total IgE ≥ 100 UI/mL were more prevalent in the wheezing group, but failed to remain in the model. Smoking during pregnancy was identified in a small number of mothers, and secondhand smoke at home was higher in the control group.

**CONCLUSIONS:**

Presence of atopy, positive Asthma Predictive Index and exposure to environmental smoke are associated to recurrent wheezing. Identifying these factors enables the adoption of preventive measures, especially for children susceptible to persistent wheezing and future asthma onset.

## RESUMO

**OBJETIVO:**

Avaliar a associação entre a sibilância recorrente e atopia, o Índice Preditivo para Asma, exposição a fatores de risco e dosagem de IgE sérica total como possíveis fatores preditores de sibilância recorrente.

**MÉTODOS:**

Estudo caso-controle com crianças de seis a 24 meses de idade atendidas em ambulatório especializado entre novembro de 2011 e março de 2013. Foram avaliados a sensibilização a antígenos inaláveis e alimentares, positividade para o Índice Preditivo para Asma e outros fatores de risco para sibilância recorrente (tabagismo durante a gravidez, presença de fumaça na residência, infecções virais e dosagem de IgE total).

**RESULTADOS:**

Foram avaliadas 113 crianças, sendo 65 lactentes sibilantes recorrentes (63,0% do sexo masculino) com média de idade de 14,8 (DP = 5,2) meses e 48 lactentes saudáveis (44,0% do sexo masculino) com média de idade de 15,2 (DP = 5,1) meses. No modelo de análise múltipla, a sensibilização a antígenos (OR = 12,45; IC95% 1,28–19,11), Índice Preditivo para Asma positivo (OR = 5,57; IC95% 2,23–7,96) e exposição à fumaça ambiental (OR = 2,63; IC95% 1,09–6,30) permaneceram como fatores de risco para sibilância. Eosinofilia ≥ 4,0% e IgE total ≥ 100 UI/mL foram mais prevalentes no grupo sibilante, mas não permaneceram no modelo. O tabagismo na gestação foi identificado em pequeno número de mães e o tabagismo domiciliar foi maior no grupo controle.

**CONCLUSÕES:**

A presença de atopia, a positividade ao Índice Preditivo para Asma e a exposição à fumaça ambiental estão associadas à sibilância recorrente. A identificação desses fatores permite a adoção de medidas preventivas, especialmente nas crianças susceptíveis à persistência de sibilância e ao surgimento de asma no futuro.

## INTRODUCTION

Wheezing resulting from peripheral airway narrowing is a frequent occurrence in the pediatric age group and may be a sign of systemic disease. Although asthma is the main cause of wheezing in infancy, this must always be a diagnosis of exclusion^16^. Many children have recurrent coughing and wheezing episodes early in life, normally during viral respiratory infections^15^. In most cases, there is a spontaneous remission of wheezing. Nevertheless, approximately 50.0% of infants and pre-school children are still wheezing at the age of six^20^. Wheezing infants are defined as children with a history of three or more wheezing episodes over a six-month period^16^.

In the International Study of Wheezing in Infants (EISL), a multi-center study to develop epidemiological knowledge of wheezing infants, prevalence of wheezing in Brazil among first-year infants varied from 43.0% to 61.0%, with 28.0% of recurrent wheezing infants^8^. This study also showed that some factors, such as early onset and number of viral infections and maternal smoking during pregnancy, had a strong correlation with cases of recurrent wheezing^18^.

The association between recurrent symptoms and personal or family history of atopy helps the early diagnosis of asthma^a^, an inflammatory disease^24^ that may lead to airway remodeling and decreased lung function in the first months of life^19^. Diagnostic criteria have been proposed for the early diagnosis of children with a high risk of developing asthma^7^. The Asthma Predictive Index (API) aims to identify wheezing infants at risk of developing the disease. Family history of atopy and atopic dermatitis are considered major criteria, while diagnosis of personal allergic rhinitis, wheezing with no upper airway infection, and blood eosinophilia ≥ 4.0% are considered minor criteria. The presence of one major or two minor criteria in an infant with three or more wheezing episodes is considered high risk for the development of asthma^7^.

Allergen sensitivity before the age of five, an important atopy marker, has been related to the development of childhood asthma^13^. However, previous studies have only investigated the prevalence of allergy in children over four years old^11^ ^,^ ^29^. The early diagnosis of asthma is essential to prevent chronic consequences of recurrent wheezing, by introducing appropriate therapy and secondary prevention. This study aims to evaluate the association between recurrent wheezing and potential predicting factors such as atopy, Asthma Predictive Index, exposure to risk factors for allergic diseases, and total serum IgE levels.

## METHODS

This is a case-control study with infants aged 6-24 months treated at the outpatient clinic of the Hospital das Clínicas of the Universidade Federal de Pernambuco (HC/UFPE) from November 2011 to March 2013. Recurrent wheezing infants from the hospital’s Allergy and Immunology Clinic composed the case group. Infants of the same age group from the Pediatrics and Childcare Clinic, with no history of wheezing, constituted the control group. The individuals were selected as they arrived for routine health care in the respective clinics.

The study enrolled children whose parents or legal guardians signed an informed consent form. The following were considered exclusion criteria: other chronic respiratory conditions, aspiration diseases, congenital anomalies, immunodeficiency disorders, prematurity, contraindication for skin testing and technical failure in blood test or collection.

Following the examination, the children’s parents or guardians answered questions based on the EISL^9^ questionnaire investigating the presence, without quantifying, of clinical parameters (airway infections, use of paracetamol and antibiotics) and risk factors related to allergic diseases (passive smoking or smoking during pregnancy, exposure to smoke – wood-burning stoves, factory chimneys or agricultural burning near home, day-care attendance, presence of dust accumulators – carpets, curtains, cushions, stuffed animals – or mold, cockroaches, and pets in the house). The Asthma Predictive Index^7^ was applied, indicating major (family history of asthma and atopic dermatitis) and minor (allergic rhinitis, peripheral eosinophilia ≥ 4.0%, and wheezing with no upper airway infection) criteria for the future onset of asthma. It was considered positive when at least one major or two minor criteria were present.

Sensitization to inhalant and food antigens was assessed by skin prick tests using standardized extracts (FDA Allergenic®, Rio de Janeiro, Brazil). The following inhalant antigens were evaluated: *Dermatophaghoides pteronyssinus*, *Dermatophaghoides farinae*, *Blomia tropicalis*, fungal mix (*Aspergillus fumigatus* and *Alternaria alternata*), dog, cat and cockroach epithelium mix (*Blattella germanica and Periplaneta americana*). Regarding food allergens, alpha-lactalbumin, beta-lactoglobulin, casein, soy, egg, and peanut were assessed. Blood was collected from infants for eosinophil count by the automated method of leukocyte differentiation in blood count and total serum IgE levels by electrochemiluminescence.

A logistic regression model was applied to the independent variables presenting p < 0.10 in the univariate analysis. The strength of the association between wheezing condition and the various outcomes was evaluated by odds ratio. Pearson’s Chi-square test was used for the categorical variables. Fisher’s exact test was used when the expected values were lower than five. Student’s t-test was used to compare the mean age between both groups. A significance level of 5% was considered.

The study was approved by the Ethics Committee for Research Involving Human Subjects of the Health Sciences Center of the Universidade Federal de Pernambuco (CEP/CCS/UFPE), CAAE registration number 0338.0.172.000-10.

## RESULTS

We evaluated 113 patients, 65 wheezing infants with a mean age of 14.8 (SD = 5.2) months and 50 controls with a mean age of 15.2 (SD = 5.1) months (p > 0.05). Two subjects from the control group were excluded due to venous access difficulties after signing the consent form and answering the questionnaire, resulting in n = 48 in this group. Males were prevalent in the wheezing group (p = 0.041). Among wheezing infants, symptoms onset age varied from one month to 18 months, with an average of 8 (SD = 5.6) months (Table 1).

**Table 1:** Characteristics of evaluated infants. Pernambuco, Northeastern Brazil, 2013.

Characteristic	Wheezing infants (n = 65)		Non-wheezing infants (n = 48)		p
	
	n/mean	%/SD	n/mean	%/SD	
Sex (male)^a^	41	63,1	21	43.8	0.041
Age in months^b^	14.8	5.2	15.2	5.1	0.701
Age of symptoms onset in months^b^	8	5.6	NA	-	-

NA: not applicable

^a^ n and %.

^b^ mean and SD.

We found sensitization to inhalant and food allergens in 11.0% and 6.0% of wheezing infants, respectively. The most prevalent inhalant allergen was *Blomia tropicalis* (6.2%) and the most prevalent food allergen was egg (3.1%). We found no combined sensitization to inhalant and food allergens in the same patient. Only one non-wheezing infant (2.1%) showed sensitization to an inhalant allergen (fungal mix) and none to food allergens (Table 2).

**Table 2:** Sensitization to inhalant and food allergens in evaluated infants. Pernambuco, Northeastern Brazil, 2013.

Allergen	Wheezing infants		Non-wheezing infants	

	n	%	n	%
Inhalant allergens	7/65	10.7	1/48	2.1
*Dermatophaghoides pteronyssinus*	2/7	28.6	0	-
*Dermatophaghoides farinae*	1/7	14.3	0	-
*Blomia tropicalis*	4/7	57.1	0	-
Fungal mix*	0	-	1/1	100
Food allergens	4/65	6.2	-	-
Cow’s milk	1/4	25.0	0	-
Egg	2/4	50.0	0	-
Peanut	1/4	25.0	0	-

* Fungal mix (*Alternaria alternata* and *Aspergillus fumigatus*).

Forty-eight percent of recurrent wheezing infants and 30.0% of non-wheezing subjects showed total serum IgE equal to or above 100 UI/mL (variation of 3 to 3.859 UI/mL) (p = 0.059) (data not shown). Peripheral blood eosinophilia greater than or equal to 4.0% of leukocytes was observed in 46.2% of recurrent wheezers and in 18.8% of infants with no previous wheezing history (p = 0.002), with an average percentage of 3.65% (SD = 1.48%; variation of 0.4% to 18.2%) (data not shown).

API was positive for 81.5% of recurrent wheezing infants and 44.8% of non-wheezers (p < 0.001). The presence of atopic dermatitis was the sole factor with no difference between both groups (Table 3).

**Table 3:** API in evaluated infants. Pernambuco, Northeastern Brazil, 2013.

API	Wheezing infants		Non-wheezing infants		p

	n	%	n	%	
Positive	53	81.5	21	43.8	< 0.001
Major criteria					
Family history of asthma	43	66.2	18	37.5	0.003
Atopic dermatitis	6	9.2	5	10.4	1.0
Minor criteria					
Allergic rhinitis	23	35.4	6	12.5	0.006
Eosinophilia ≥ 4.0%	30	46.2	9	18.8	0.002
Wheezing without URI	35	53.9	NA		-

API: Asthma Predictive Index; URI: upper respiratory tract infection; NA: not applicable

Among the risk factors investigated, only the presence of pets in the house and exposure to smoke caused by environmental pollution were individually associated with the occurrence of wheezing (Table 4).

**Table 4:** Risk factors associated to recurrent wheezing in evaluated infants. Pernambuco, Northeastern Brazil, 2013.

Risk factor	Wheezing infants		Non-wheezing infants		p

	n	%	n	%	
Day care attendance	8	12.3	7	14.6	0.725
Dust accumulators	53	81.4	41	85.4	0.586
Mold at home	27	41.5	21	43.8	0.814
Cockroaches at home	46	70.8	36	75.0	0.618
Pets	18	27.7	22	45.8	0.046
Smoking during pregnancy	6	9.2	5	10.4	> 0.999
Passive smoking	17	26.2	19	39.6	0.130
Exposure to smoke	39	60.0	17	35.4	0.010
Caesarean section	36	55.4	20	41.7	0.149
EB ≥ 6 months	19	29.2	20	41.7	0.169
Repeated URI	48	73.8	31	64.6	0.289

EB: exclusive breastfeeding; URI: upper respiratory tract infection

In the multiple analysis model, sensitization to allergens (OR = 12.45; 95%CI 1.28–19.11), positive API (OR = 5.57; 95%CI 2.23–7.96) and exposure to smoke (OR = 2.63; 95%CI 1.09–6.30) persisted as important risk factors for wheezing (Table 5). We excluded variables that could adjust the model based on previous knowledge, such as smoking during pregnancy, passive smoking after birth, upper airway infections, total IgE, and day care attendance^4^ ^,^ ^22^, since their p-value was greater than 1.

**Table 5:** Multiple analysis of factors associated with wheezing in evaluated infants. Pernambuco, Northeastern Brazil, 2013.

Associated factor	OR	95%CI	p
Sensitization to allergens	12.45	1.28–19.11	0.029
Positive API	5.57	2.23–7.96	< 0.001
Exposure to smoke	2.63	1.09–6.30	0.030

API: Asthma Predictive Index

## DISCUSSION

In this study evaluating infants aged 6-24 months, sensitization to inhalant and food allergens, positive Asthma Predictive Index and exposure to smoke were associated with recurrent wheezing.

Longitudinal studies established early sensitization to allergens as one of the main risk factors for persistent wheezing^25^ ^,^ ^28^, sensitization to inhalant household allergens being the most important^8^. In addition, asthma severity is increased in atopic patients exposed to high levels of allergens^17^.

Similarly to this study, several other authors have observed a greater prevalence of wheezing among male infants, an intrinsic risk factor. The average age for the onset of symptoms in Brazil in the EISL was five months^18^. In our study, the average age was slightly higher.

In Germany, a multicenter study assessing 1,290 children with a family history of atopy investigated the relation between the presence of IgE specific to inhalant and food allergens at 12 months and the development of atopic disease at the age of six. The authors observed that children with early sensitization to inhalant allergens presented greater risk of developing allergic disease^6^.

A study carried out in different Brazilian localities observed that sensitization to food was prevalent in the first years of life, and to inhalants in older age groups^23^. These findings were also observed in other countries^2^ ^,^ ^27^, but not in this study. In this case, sensitization to the house dust mite was prevalent, with *Blomia tropicalis* being the main allergen. The high levels of sensitization to mites in Brazil may be associated to the high level of exposure to these allergens at home^26^. The incidence of specific sensitization to the house dust mite tends to increase progressively with age, while sensitization to food decreases^11^. However, the design of this study does not allow us to reach such an assessment.

Total serum IgE and peripheral blood eosinophilia^3^ ^,^ ^19^ have been associated to a persistent wheezing condition. Similarly to Naspitz et al.^23^, we also observed a wide variation in total IgE levels, the highest occurring in the case group (p = 0.059). Medeiros et al.^21^ concluded that total serum IgE can be influenced by current or past presence of intestinal parasites. Peripheral blood eosinophilia count was also higher in the group of wheezing infants (p = 0.002).

In this study, infants with positive API showed a higher probability of persistent wheezing. This outcome was expected, since this scoring system was developed to identify among recurrent wheezing infants those with greater risk to continue wheezing until school age^7^. We observed, however, two recurrent wheezing infants with positive skin tests but negative API, which might suggest a limitation of this index.

Among avoidable risk factors, exposure to smoke from environmental pollution was most strongly associated with the occurrence of wheezing. Children exposed to smoke were 2.6 times more likely to be recurrent wheezers than children who had no contact with smoke. Indeed, air pollution, whether from the use of biomass fuels (wood, coal, animal manure, among others) or from vehicles, increases the risk and severity of asthma^a^. Environmental tobacco smoke and or smoking during pregnancy were not identified as risks factors in this study. This may be due to the low prevalence of smoking during pregnancy and the fact that passive smoking was more prevalent in the control group. Nevertheless, exposure to tobacco smoke must be avoided.

The association between viral respiratory infection and atopy, despite still being controversial, has also been considered a risk factor for persistent wheezing and later asthma onset^15^. In this study, prevalence of viral infection was high in both groups, although there was no significant difference between them.

Reduced inflammatory response or non-exposure to allergens may have led to the higher percentage of negative results in the immediate hypersensitivity test^14^. This may have affected our findings. A further limitation concerns data interpretation, since no stool and specific anti-ascaris IgE tests were carried out, which would be necessary to eliminate any present or past geohelmynth infection that could alter the peripheral blood eosinophilia count, total serum IgE levels, and positive response to immediate allergen hypersensitivity^1^.

Fitzpatrick et al.^12^ showed the importance of atopy in defining pediatric asthma. Atopy can be confirmed by detecting allergen-specific IgE by *in vivo* immediate hypersensitivity skin testing^23^.

The higher frequency of positive reaction to allergy skin tests in wheezing infants suggests that early sensitization influences recurrent wheezing. Such findings suggest that immediate response skin tests, which present good sensitivity, specificity and safety, should be carried out when assessing wheezing infants, alongside API assessment.

The identification of allergen sensitivity allows asthma diagnoses in infants and pre-school children to be guided by more objective parameters, supporting the adoption of drug treatment and directing environmental control measures, thus reducing allergen exposure by children susceptible to persistent wheezing. Early asthma diagnosis and treatment in these children, alongside objective measures to prevent exposure, improve their quality of life and the prognosis of asthma^a^. This context enables the development of studies to ascertain the atopic profile and assess the routine use of skin testing in early wheezing infants.

## Footnotes

a Global Initiative for Asthma. Global Strategy for the diagnosis and management of asthma in children 5 years and younger. Update 2009 [cited 2013 nov 25]. Available from: http://www.ginasthma.org/local/uploads/files/GINA_Under5_2009_CorxAug11.pdf
